# A new database of the analysis of the physiological needs in amateur female basketball during official matches

**DOI:** 10.1038/s41597-023-02747-2

**Published:** 2023-12-01

**Authors:** Abraham Batalla-Gavalda, Raul Montoliu, Jose Vicente Beltrán-Garrido, Francesc Corbi

**Affiliations:** 1 EUSES Escola Universitária de la Salut i l’Esport, Rovira i Virgili University, 43870 Tarragona, Spain; 2 Grup de Recerca en Ciéncies de l’Esport INEFC Barcelona, 2021 SGR 01191, Institut Nacional d’Educació Física de Catalunya (INEFC), Universitat de Barcelona, Barcelona, Spain; 3 Institute of new imaging technologies (INIT), Jaume I University, Castellon de la Plana, Castellón Spain; 4 Department of Education Science, School of Humanities and Communication Sciences, Universidad Cardenal Herrera-CEU, CEU Universities, Calle Grecia 31, 12006 Castellon de la Plana, Spain; 5 Institut Nacional d’Educació Física de Cataluña, Facultat de Lleida, Universitat de Lleida, 25192, Complexe de La Caparrella, s/n, Lleida, Spain

**Keywords:** Physiology, Social sciences

## Abstract

The PHYAFB database is a valuable resource for studying the physiological demands of female amateur basketball players during high-stress official games. It contains heart rate data from ten players aged 18 to 26, collected during ten crucial relegation phase matches, with 348,232 HR samples in CSV and Excel formats for easy access and analysis. The database includes *Python* source code for initial examination. The primary aim of the PHYAFB database is to provide a useful reference for other teams facing similar situations. Furthermore, the database represents a unique and valuable resource for sports scientists, coaches, and trainers seeking to comprehend the physiological demands of female basketball players during official competitions. Through the analysis of heart rate data, coaches and trainers can identify the intensity and duration of physical activity during games, enabling the development of more effective training programs. Additionally, the database can be used to compare the physiological demands placed on male and female basketball players or to investigate the impact of different game strategies on player performance.

## Background & Summary

Characterised by collaborative teamwork and competitive opposition, basketball exhibits dynamic intensity fluctuations that elicit continuous changes in heart rate (HR)^1^. As suggested by Abdelkrim *et al*. in^1,2^, these fluctuations may reach maximum levels, classifying basketball as an intermittent high-intensity sport^3^. The analysis of basketball performance has traditionally employed the concept of internal load (IL)^4^. IL refers to the physiological effect exerted on the body of an athlete in response to the prescribed task, known as external load (EL)^5^. The IL is influenced by the level of EL and its individual impact on each athlete’s body, as well as additional factors such as training level and genetic potential^6^.

Various indicators have been employed to assess internal load (IL) in basketball. These indicators encompass the analysis of HR dynamics^7,8^, Rating Perceived Exertion (RPE)^9,10^, as well as their interrelationship^11^. Furthermore, the hormonal profile, specifically the testosterone-cortisol ratio^12,13^, and the concentration of metabolites such as lactate or ammonium^14,15^, have also been examined. Among these indicators, HR has predominantly been utilised as a straightforward parameter to analyse the physiological demands in team sports due to its simplicity^16,17^. Utilising a chest sensor for heart rate monitoring proves to be a more comfortable and advantageous approach for both athletes and researchers when compared to alternative, invasive methods of assessing internal load, like hormonal analysis involving testosterone and cortisol^18^. Furthermore, the chest sensor emerges as the most efficacious means for maintaining real-time vigilance over internal load, promptly capturing fluctuations in intensity^19^. Unlike invasive techniques that require blood extraction, a heart rate sensor simply needs to be worn or attached to the body, causing no discomfort or inconvenience during exercise. Moreover, heart rate sensors are widely accessible and affordable, making them a practical choice for determining the intensity levels achieved during sports participation^20^.

Regarding basketball, a multitude of studies since the 1990s have examined the physiological demands of the sport, particularly using heart rate as a prominent indicator^21–32^, but none of them made publicly available the data used in those studies. There are also some studies that focus on female basketball during official competitions^23,26,29,33–39^. For instance, Rodríguez-Alonso *et al*.^39^ analysed a professional Spanish first-division women’s basketball team. Another significant study is the research conducted by Matthew *et al*.^23^, which examined heart rate patterns in a women’s basketball team competing at the British University Sport Association (BUSA) Premier League. Additionally, Scanlan *et al*.^26^ investigated the physiological demands during eight official matches of a professional team from the Australian first division. A recent standout publication by Sanders *et al*.^29^ focused on heart rate analysis at various levels of intensity in an NCAA Division I women’s basketball team. The study revealed that players spent a significant portion of the game time above 85% of their maximum heart rate (HRmax), with the final period exhibiting the highest intensity. Unfortunately, the previously mentioned studies did not publicly share the data used in their research.

Several factors contribute to the lack of publications examining the physiological demands of amateur female basketball players during official competitions. Firstly, accessing an amateur club for scientific research and implementing methodologically sound protocols can be challenging. Secondly, establishing a close relationship with the team’s coaching staff is crucial to mitigate potential disruptions to the players’ performance resulting from research intrusions into pre-competition protocols. Thirdly, the risk of a serious injury that could lead to the loss of the sample poses a concern for longitudinal analyses of the same team. Lastly, the logistical difficulties associated with measuring and recording competitions in various facilities where amateur matches take place add further complexity to the research process. Although these difficulties can be less important when working with professional teams, some of them can be also present. For instance, the absence of data on particular players due to injuries, the coach’s decision, etc. Furthermore, it is important to note that conducting a project of this magnitude is subject to the regulations of the specific competition. In our case, we had to adhere to the regulations set by the organising federation, as the use of heart rate monitors during the competition was not initially permitted. We made official requests to the federation, providing an informative document and seeking permission from the rival clubs involved in the study, as well as the relevant councils, to gain access to their facilities. We focused on amateur sports since the amount of possible recipients of this kind of research is bigger just for the large existing number of amateur teams.

This paper introduces the *PHYAFB* database^1^, which comprises heart rate data obtained from ten female players aged 18 to 26 years. The data was collected during ten intense matches that took place during a critical relegation phase. The database is stored in CSV and Excel formats, ensuring convenient access and facilitating analysis. Additionally, the database is accompanied by Python source code to assist in the initial exploration and examination of the data. In total, 348,232 HR measurements are included in the dataset.

Given the aforementioned background and the challenges associated with accessing and controlling an official amateur sports competition, we believe that this database can serve as a valuable tool for sports analysts, coaches, physical trainers, and researchers interested in studying physiological variables in small field team sports. To the best of our knowledge, no such databases exist in the literature, making the database presented in this article a novelty in this research field. The data presented in these files can be utilised for statistical analyses, enabling the improvement of sports performance and the study of trends or patterns of play, considering the physiological behaviour based on possession type or score differentials. Furthermore, with the implementation of advanced statistics, it becomes possible to generate predictive models and even simulate player behaviour, leading to adjustments in season planning and game strategies.

Regarding the publications derived from this database, we have achieved significant findings. One publication explores the relationship between heart rate and subjective perception of effort^11^, while another publication focuses on heart rate variations based on score differentials^40^. These publications are just a glimpse of the valuable insights that can be derived from the database.

## Methods

### Participants and ethical requirements

The study sample comprised 10 amateur women’s basketball players competing at the highest level within Catalonia, specifically in the context of the Catalonia Cup in Spain. Table 1 shows the anthropometric data of each player together with age, body fat percentage, the maximum achieved HR during games, and the playing position. During the study, special attention was paid to ensure that the participants did not perform a physical activity of moderate or high intensity outside of the assessment or training sessions. In addition, the participants had no personal or family history of heart disease, nor had they suffered any injury that could alter regular sports practice in the 6 months before the study.

**Table 1:** Anthropometric data, age, max HR, body weight and playing position of the ten players who participate in the study.

Player	Playing position	Age	Height (cm)	Body weight (kg)	Max. HR (ppm)	Body fat (%)
1	F	21	175	71.7	195	23.2
2	F	26	170	64.5	197	20.1
3	G	24	168	50.7	199	14.9
4	G	21	167	67.2	205	23.7
5	F	18	184	65	195	19.5
6	F	18	182	67.8	200	22.7
7	F	19	179	65.5	205	16.8
8	C	24	188	73.8	205	18.5
9	F	22	180	66.6	192	21.3
10	C	20	185	95.6	197	26.7
Mean		21.3	177.8	68.84	199	20.74
Std		2.71	7.45	11.21	4.69	3.51

Mean and standard deviation are also provided. *G*, *F* and *C* stand for Guard, Forward and Center, respectively.

None of the study participants received a financial or in-kind reward for their collaboration. Likewise, they signed informed consent and a protocol was established to deliver and explain the results. At the time of the study, none of the participants was taking any type of medication, did not follow a diet, and did not suffer from respiratory or metabolic alterations.

The study was approved by the ethics committee of the *Consell Cataláde l’Esport* (025-CEICGC-2022). This study was carried out taking into account the principles of the Declaration of Helsinki for research with human beings^41^, as well as the criteria established in the Biomedical Research Law (Law 14/2007, of July 3, on Research Biomédica, 2007) and in accordance with the Data Protection Law (BOE.es - BOE-A-2018-16673 Organic Law 3/2018, of December 5, Protection of Personal Data and guarantee of digital rights).

### Acquisition setup

The players performed 3 training sessions per week of 2 hours duration and one game on the weekend, Half of the games were played at home and the other half away, so a trip was necessary to reach the rival team’s court. Before recording the heart rate in competition, a friendly match was organised the week before data collection, so that the participants and the research team could familiarise themselves with the data collection protocol. Subsequently, the last ten official matches of the relegation phase were analysed. HR monitoring during matches was obtained using Suunto Team Pack^TM^ heart rate monitors and the information was collected in real-time by the Suunto Team Pod^TM^ unit. Heart rate monitors were placed 10 minutes before the start of warm-up. All the participants were instructed to place the heart rate monitors correctly before conducting the study. At the same time, the matches were recorded with 2 video cameras (JVC-GZ620SE HDD. Hong Kong, China) synchronised with the heart rate monitors by means of an acoustic and visual signal, just before the start of the warm-up. The synchronisation was repeated at the beginning and end of each quarter. The cameras were placed in an elevated position on the grandstand from which it was possible to record at least half a track without being moved. Video analysis was performed second by second, using frame-by-frame recording, with a precision of 0.04 s and a recording frequency of 25 Hz.

The Suunto Team Pod^TM^ heart rate monitors and the camcorders were purchased 2 weeks before the start of the project. The Suunto Team Pod^TM^ has the following specifications: width 50 *mm*, length 171 *mm*, height 75 *mm*, weight 180 g (including the tripod adapter and antenna), operation range of up to 100 *m*, sampling frequency of 1 *Hz* (i.e. one HR measurement each second) and power consumption of approximately 50 *mA*. The Suunto Team Pod^TM^ heart rate monitor has been widely used in previous works^42–47^ and, according to Montgomery *et al*.^48^, it has shown to be reliable, with a typical error of $0.64\frac{mL}{kg\cdot min}$ (≈1.5%) and a coefficient of variation of (6%) when estimating the aerobic (*V*_*o*2_) energy demands obtained from the HR reading provided by this particular model of heart rate monitor.

### Sensor placement

**Fig. 1:**
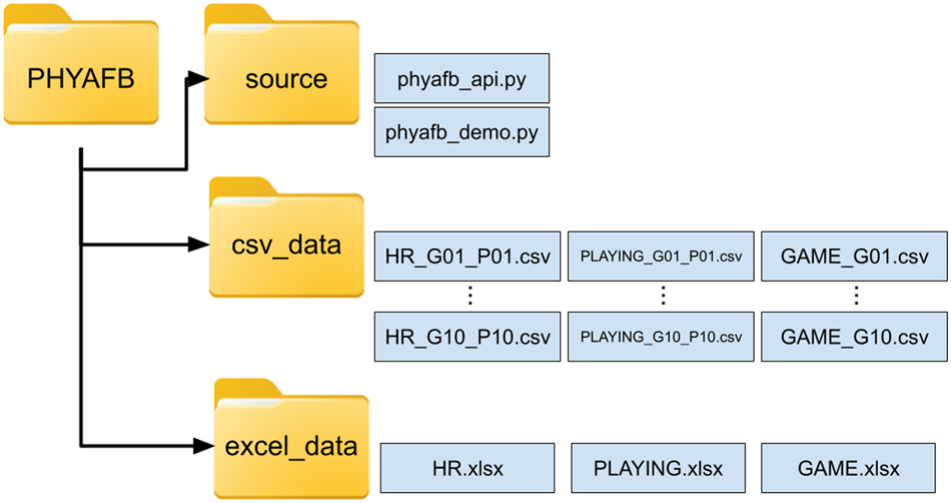
Two cameras were placed at the stands, where each camera covered half of the court. The sensor for receiving data from the pulsometer was located close to half of the court. Players wore the pulsometer in the chest.

The heart rate monitors were located on the chest (see Figure 1), just below the sports bra. To prevent the heart rate monitors from moving during the match, pieces of Velcro were sewn to the tops of the players, better fixing the position of the chest band. The receiving antenna for the heart rate monitor signals (Suunto Team Pod^TM^) was located in the stands, at the height of the middle of the track, facilitating signal reception from all areas of the track. Finally, the recording cameras were located in the stands, in a raised and fixed position, to record half a track with each of them. The subsequent synchronisation of the images was performed with the Kinovea program (version 0.8.24).

### Data processing

After recording the matches, the heart rate (HR) data captured using the Suunto Team Pack^TM^ program was downloaded in CSV format. The recorded data had a frequency of 1 Hz, providing HR values for each second of the game. To ensure accurate synchronisation between the HR data and the on-court events, the recorded videos were carefully reviewed. For every second of the footage, the researchers manually matched the HR data with the corresponding court conditions, including whether the player was on the court, whether the ball was in play, and whether the team was in the attacking or defending phase. This meticulous process allowed for precise alignment of the HR data with the game dynamics and actions on the court.

## Data Records

**Fig. 2:**
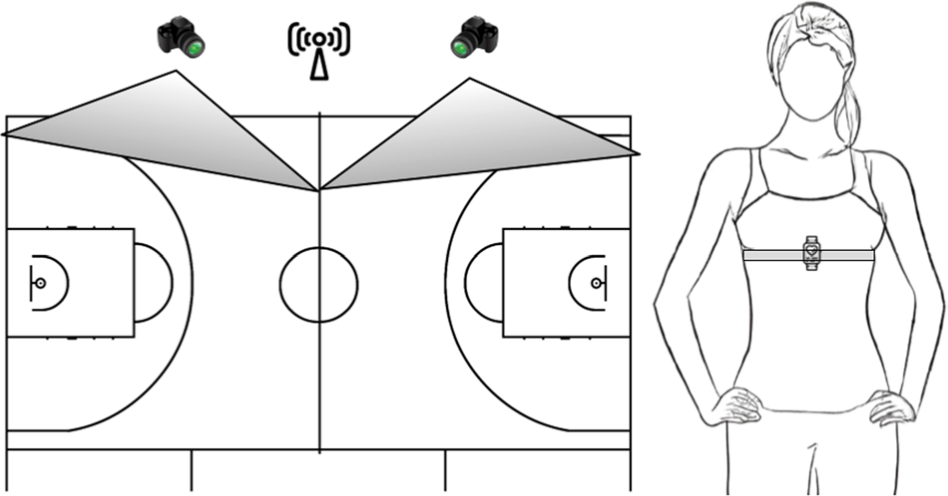
Folders and files structure of the *PHYAFB* dataset.

The dataset can be found at https://zenodo.org/records/10054915 webpage^1^. Fig. 2 shows the structure of the dataset. The *source* folder contains the included source code, explained in the corresponding Section. The *csv_data* folder contains the data files which consist of three types of files: the *HR* files, the *Playing* files, and the *Games* files. Each file type is extensively described in the subsequent sections. All the data files are in CSV format, with the first row serving as the header row, specifying the field names for easy reference and understanding. We have also provided Excel files within the *excel_data* folder. Specifically, there is one Excel file dedicated to HR data, featuring separate sheets for each game displaying the HR data of all players. Similarly, we have included a distinct Excel file for information about whether the players were playing or not. Lastly, an Excel file containing game information is also available. We’ve included both data file types to ensure easy access for a diverse range of researchers to the data in this study.

Table 2 presents the duration of each game included in the studio, along with the corresponding number of heart rate (HR) measurements obtained for each player. It should be noted that each second during a game corresponds to an HR measurement. On average, a match lasts for 5,192.7 seconds (with a standard deviation of 228.92 seconds). It is important to highlight that an official match typically lasts for 40 minutes, equivalent to 2,400 seconds. Hence, on average, each match has an additional 2,661.2 seconds where the clock was not running.

**Table 2:** Number of seconds of each game.

Game	Number of samples
1	4793
2	5062
3	5171
4	5081
5	4956
6	3945
7	5234
8	5504
9	5493
10	5373
Mean	5061.2
Std	453.50

This number also corresponds to the number of HR samples obtained for each player at each game. For Game 6 only the three first quarters were recorded. For estimating the mean, the number of seconds of game 6 has been multiplied by 4/3.

Furthermore, it is worth mentioning that for every game, the *HR* and *Playing* files for all participating players, as well as the corresponding *Game* file, contain an equal number of rows representing seconds and HR samples.

### HR files

In these files, the heart rate of each player at each game is shown. The filename of these files is *HR_Ggg_Ppp.csv*, where $pp\in [1,10]$ is the identifier of the player, and $gg\in [1,10]$ is the identifier of the game. Therefore, the file *HR_G05_P03.csv* shows the HR of the player with id 3, at each second during the 5th game. The file has two columns, the first one is “TIME” the time (in seconds) when the HR data was recorded and the second “HR” is the HR of the player in this time period. Table 3 shows the first 15 rows of the file *HR_G05_P03.csv*. When a player did not play a particular game, no file was generated.

**Table 3:** 15 first rows of the file *HR_G05_P03.csv*.

TIME	HR
1	101
2	100
3	99
4	98
5	98
6	98
7	98
8	98
9	98
10	99
11	99
12	99
13	99
14	99
15	100

The *HR.xlsx* file, included in *excel_data* folder, presents the same information but in a unique Excel file. Each sheet of this file shows the HR measurements for all players.

### Playing files

These files are useful to know when the player was on the court playing each game. When the player was not playing, she was sitting on the bench. The filename of these files is *PLAYING_Ggg_Ppp.csv*, where $pp\in [1,10]$ is the id of the player, and $gg\in [1,10]$ is the id of the game. Therefore, the file *PLAYING_G05_P03.csv* shows if the player with id 3 was on the court during the 5th game. The file has two columns, being the first one “TIME” the time (in second) of the match and the second “PLAYING” is 1 if the player was playing on the court and 0 if not (she was on the bench). Table 4 shows the first 15 rows of the file *PLAYING_G05_P03.csv* showing that the player was not on the court during the very initial moment of the game. When a player did not play a particular game, no file was generated.

**Table 4:** 15 first rows of the file *PLAYING_G05_P03.csv*.

TIME	PLAYING
1	0
2	0
3	0
4	0
5	0
6	0
7	0
8	0
9	0
10	0
11	0
12	0
13	0
14	0
15	0

Like the *HR.xlsx* file, we have also provided the *Playing.xlsx* file in the *excel_data* folder. This file consolidates the same information into a single Excel document. Each sheet within this file displays whether the players were on the court during the respective games.

### Game files

This file shows, for each second of the game, an analysis of what happened in this particular time moment has been performed. The filename of these files is *GAME_Ggg.csv*, where $gg\in [1,10]$ is the id of the game. The file has the following fields:

- TIME: Time in seconds
- QUARTER: 1 to 4, meaning the quarter number and 0 meaning that the game is in the intervals between quarters.
- ANALYSIS: This field has one of the next possible values:
  - 0: Rest intervals.
  - 1: The clock is on.
  - 2: The clock is off and there is not a Time out or a Free Throw situation.
  - 3: The clock is off and there is Time out.
  - 4: The clock is off and there is a Free Throw situation.

- ATTACK_DEF: 1 when the clock is on and the team is attacking, 2 when the clock is on and the team is defending and 0 otherwise (i.e. the clock is off).
- OWN_POINTS: The number of points of the team participating in this study.
- OPP_POINTS: The number of points of the opponent team.

Table 5 shows the first 15 rows of the file *GAME_01.csv*. It corresponds to the initial seconds of the game when the team was defending and none of the teams had scored yet. In the last two rows, there is a change of possession and the team starts to attack.

**Table 5:** 15 first rows of the file *GAME_01.csv*.

TIME	QUARTER	ANALYSIS	ATTACK_DEF	OWN_POINTS	OPP_POINTS
1	0	0	0	0	0
2	1	1	2	0	0
3	1	1	2	0	0
4	1	1	2	0	0
5	1	1	2	0	0
6	1	1	2	0	0
7	1	1	2	0	0
8	1	1	2	0	0
9	1	1	2	0	0
10	1	1	2	0	0
11	1	1	2	0	0
12	1	1	2	0	0
13	1	1	2	0	0
14	1	1	1	0	0
15	1	1	1	0	0

The file *Game.xlsx*, included in the folder *excel_data*, presents the same information in a unique Excel file.

### Missing data

In relation to the lost data, we can indicate that on 29 occasions of the 100 that could be obtained at most (There is 10 matches and 10 players), it was not possible to obtain the data. These data losses were determined, firstly, by the injuries of the players during the data collection process, and secondly, by the non-attendance of the matches for personal reasons. It is important to note that the players were amateurs, and that means that some players had to miss games for work reasons or for university academic reasons. In those games, other players not participating in this study played the games. Table 6 shows which players played each game, together with the total amount of players in each game (last column) and the total amount of games played in each game (last row). Unfortunately, in none of the games, the total players participating in the study played the game. Only the players with ID 3 and 5 played the 10 games.

**Table 6:** This table shows (using a ✓) which player played which game.

Game	Player ID										
	1	2	3	4	5	6	7	8	9	10	Total
1	✓	✓	✓		✓	✓	✓	✓	✓		8
2	✓	✓	✓		✓		✓	✓	✓	✓	8
3	✓	✓	✓		✓		✓	✓	✓		7
4	✓	✓	✓	✓	✓		✓	✓	✓		8
5		✓	✓		✓	✓	✓	✓	✓		7
6	✓		✓		✓	✓	✓	✓			6
7	✓		✓		✓		✓	✓			5
8	✓	✓	✓	✓	✓	✓		✓	✓		8
9	✓	✓	✓		✓		✓		✓	✓	7
10	✓	✓	✓		✓	✓	✓	✓			7
Total	9	8	10	2	10	5	9	9	7	2	

The last row shows the number of games played by each player. The last column shows how many games are played by each player.

In addition, For the Game 6, the last quarter of the game was not recorded.

## Technical Validation

This section presents a preliminary study aimed at providing insights into the potential types of analyses that can be conducted using the proposed dataset and provided source code.

**Fig. 3:**
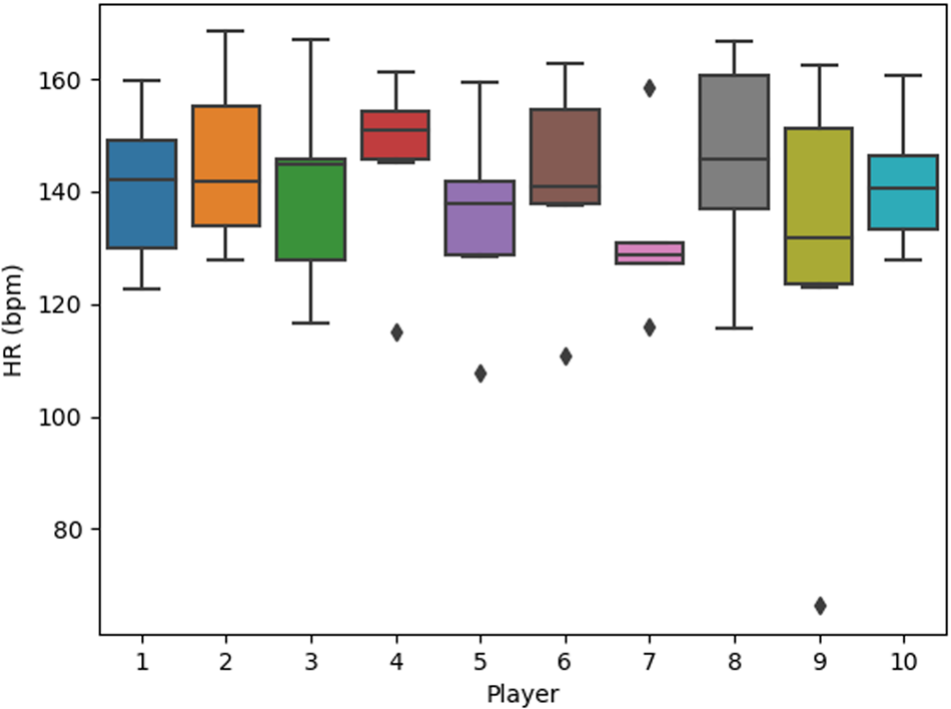
Mean HR of the players across the played games.

In Fig. 3, the average heart rate (HR) of individual players during the games they participated in is depicted. Each column represents a different player. The duration of the entire game, from start to finish, has been considered for each player. This duration is referred to as the Whole Game (WG), encompassing both the time the player spends on the court and the time spent on the bench. While a basketball game typically lasts for 40 minutes of live time, the total duration of a typical match can extend up to 120 minutes. As a result, players experience periods of elevated HR during active gameplay, alternating with periods when the clock is stopped or when the player is on the bench. Consequently, the average HR tends not to be very high, as illustrated in Fig. 3.

**Fig. 4:**
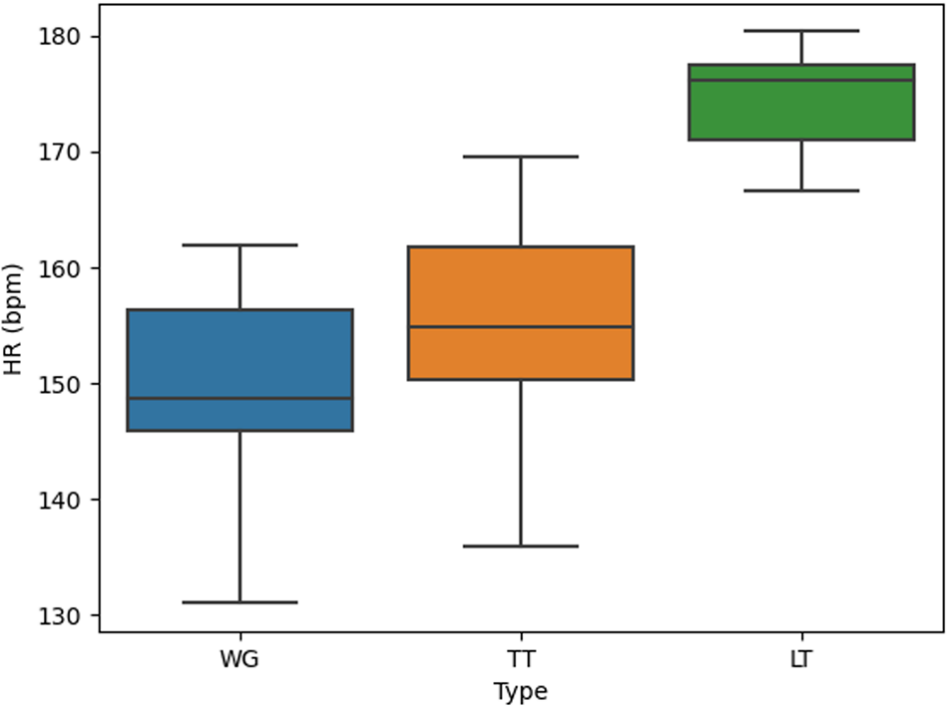
Average HR of player 1 across the played games using the time classification in Whole Game (WG), Total Time (TT) and Live Time (LT) proposed by McInnes *et al*. in^21^.

Figure 4 provides a comparison of the average heart rate (HR) for a specific player (identified as Player 1) using three different methods of calculating player participation: a) Whole Game (WG), b) Total Time (TT), and c) Live Time (LT). In the WG approach, the complete duration of the match is considered, encompassing the time when the player is both on the court and on the bench. TT takes into account the player’s on-court time, including instances when the clock is running as well as when it is stopped (e.g., during free throws or timeout situations). On the other hand, LT focuses solely on the player’s on-court time while the clock is actively running. As depicted in Fig. 4, the average HR observed in the TT method tends to be higher than that in the WG method, indicating that the inclusion of off-court time periods leads to a lower average HR. Furthermore, the average HR in the LT method is even higher than that in the TT method, suggesting that by considering only on-court time during active play, the HR levels tend to be more elevated. This behaviour is the same for the rest of the players of the team (not shown in this paper).

**Fig. 5:**
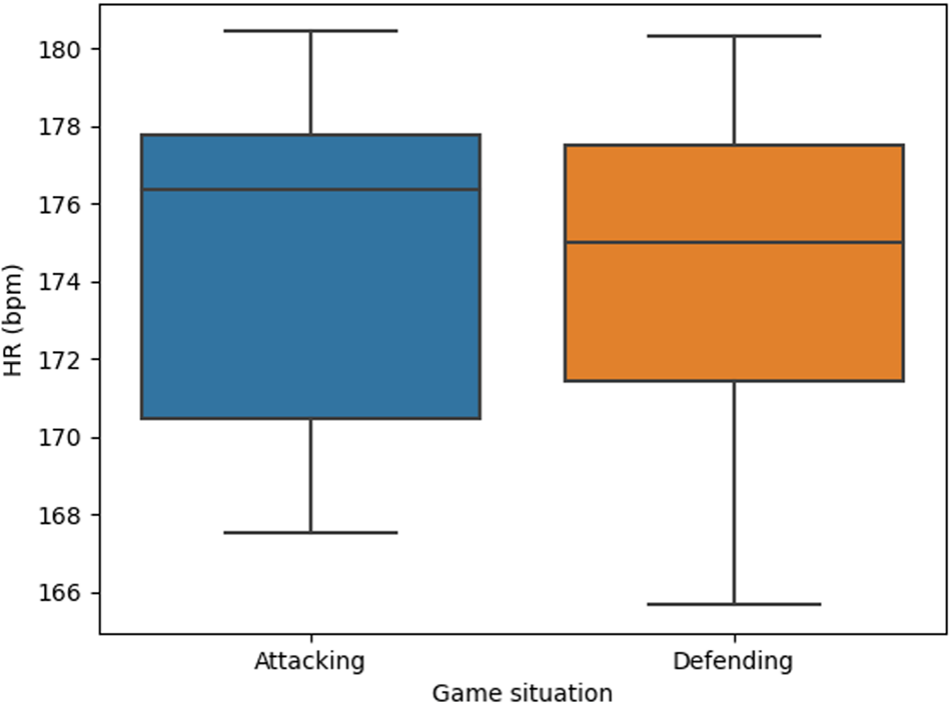
Average HR of player 1 across the played games when the player was attacking versus when she was defending.

Figure 5 displays the average heart rate (HR) for a specific player (Player 1) during two distinct game situations: when the team is attacking and when the team is defending. The purpose of this analysis is to examine potential differences between these two scenarios. However, the study reveals that no significant differences can be observed between the player’s HR in attacking and defending situations. This outcome can be interpreted as positive news, as it suggests that the player demonstrates a consistent level of commitment and effort regardless of whether the team is in an attacking or defending position. The player’s dedication remains steady, contributing equally to both aspects of the game. The data suggest that the player’s level of effort is quite similar in both situations, leading to comparable HR levels. It is worth noting that this pattern holds true not only for Player 1 but also for the remaining players on the team, as observed in the analysis of the entire team (not included in this paper).

In Figs. 3–5 the box plots provide a visual representation of the data distribution. The whiskers in these plots signify the minimum and maximum values, with the box’s edge closest to zero representing the 25th percentile. The black line within the box represents the median, and the boundary of the box farthest from zero indicates the 75th percentile. Any data points falling outside the whiskers, represented by diamonds, are considered outliers.

## Data Availability

A Python source code is provided to help future dataset users. We provide an API for accessing the data and some examples of the type of studies that can be done by using this dataset. The API (*phyafb_api.py* file) is composed of the following functions: • *read_hr_file*: This function is designed to read an HR file for a specific game and player. It returns a list containing the HR values recorded. If there is no file available for the given game and player (indicating that the player did not participate in that particular game), the function returns an empty list. • *read_playing_file*: The purpose of this function is to read a PLAYING file for a specific game and player. It returns a list consisting of binary values (1 or 0). A value of 1 signifies that the player was on the court at that particular moment, while a value of 0 indicates that the player was on the bench. If there is no file available for the given game and player, the function returns an empty list. • *read_game_file*: This function facilitates access to specific fields within the GAME files. Along with the game and player parameters, the field name needs to be specified as an additional parameter. The function then returns a list containing the values of the specified field. If there is no file available for the given game and player, the function returns an empty list. We also provide a Python script *phyafb_demo.py* to obtain the figures shown in the previous section.
